# Clinical Studies for Nurses: For Second and Third Year Pupil Nurses

**Published:** 1910-02

**Authors:** 


**Clinical Studies for Nurses:** For Second and Third Year Pupil Nurses. By **Charlotte A. Aikens,** formerly Superintendent of Columbia Hospital, Pittsbury, and of Iowa Methodist Hospital, Des Moines. 12 mo of 510 pages, illustrated. Philadelphia and London: W. B. Saunders Company. 1909. (Cloth, $2.00 net.)

This work is on similar lines as "Primary Studies for Nurses," and is the second book of a series. The aim has been to include the main points about common diseases and their management which the nurse should know during the second year of her training in order that she may understand how to intelligently assist the physician and care for the sick under his direction. The work is well illustrated and the arrangement good. A series of six-hundred questions for review is a valuable feature.

